# The Hospital Nursing Supplement

**Published:** 1893-09-30

**Authors:** 


### "The Hospital" Nursing Mirror.

Being the Extra Nursing Supplement of "The Hospital" Newspaper.

[Contributions for this Supplement should be addressed to the Editor, The Hospital, 428, Strand, London, W.C., and should have the word "Nursing" plainly written in left-hand top corner of the envelope.]

### News from the Nursing World.

### PENSION AND SICK ASSURANCE.

"I never get really ill, so I don't see the fun of paying into a sick fund," is a superficial way of dismissing this subject. There are, indeed, a few happy people left, who really enjoy good health, and seem secure from the ills which afflict their less fortunate companions. Still, nobody can expect altogether to escape from perils, such as infection or casualties. A time must come when the bravest and strongest of workers may find with alarm that she is laid aside for some weeks, and that everything is "going out" and nothing "coming in." One of the nurses now beginning to look wisely ahead, paid into the Fund for Sickness Assurance, that valuable branch of the Royal National Pension Fund, her first quarterly premium on September 1st. On the 15th she contracted scarlet fever, and on the 21st satisfactory medical evidence was received showing that she would be for some weeks to come in, an isolation hospital. The sick pay of the nurse was immediately forwarded reckoned from the date of her being incapacitated for work. Her illness will be easier borne with the knowledge that consideration as to "ways and means" need not add to its burden.

### OUR CHRISTMAS COMPETITIONS.

Many readers are just completing, or have already completed their holidays, and so are able, with restored health and energy, to turn their attention to the daily round. It is most desirable that a much larger number than ever before should enter for this competition. We anticipate that an immense variety of garments will this year be made by our readers for the adult-sick poor who have to spend their Christmas Day within hospital walls. We find that the parcels which are the result of our annual competition receive such hearty welcomes at the institutions to which they are sent, that we hope to see them increase in bulk and in number each year. The prizes will be: (1) For the best pair of socks knitted by a nurse, 5s.; (2) for the best pair knitted by any Hospital reader, 5s.; (3) for the best made flannel shirt, 10s.; (4) for the best flannel or flannelette bed-jacket, 10s.; (5) for the best flannel petticoat, 10s.; (6) for the best made and simplest shaped dressing gown made and cut out by a nurse, 20s. Long seams may be done by machine. With the view of enabling an estimate to be formed of the progress made, we shall be glad to receive the names of competitors, and to hear which of the six prizes each will enter for.

### NOTES AND QUERIES.

In the beginning of this year readers of The Hospital had their attention specially called to the dimensions to which its correspondence department had attained. Their kindly co-operation was invited and secured, but it is perhaps impossible for the reading public to understand the pressure of the extraneous work brought to bear upon those who write for them. Things that make no show often take more time than those which are patent to every one. Any worker understanding this can also comprehend the advantages of intelligent co-operation with an editor. For instance, why are not all queries put shortly and simply, with the full name and address of the questioner attached? Again, letters on all subjects intended for publication should be short. If the writer of a lengthy epistle saw the number of similar communications in the editor's box, a speedy attempt to reduce the quantity, and to improve the quality of each literary production would surely result. Short letters are generally cleverer, as well as more readable, than long ones. Just as good essence is better than largely diluted mixtures. Kindly co-operation has invariably attended each "new departure" of the editor's, and a little forethought on the part of Hospital readers will secure intelligent consideration from his numerous correspondents.

### IS IT WORTH DOING?

The words "sanitary science" are constantly brought forward in some connection or other, and a little knowledge on that large subject is not difficult of attainment. For some time a movement in favour of the appointment of women inspectors has been anticipated, and The Hospital has tried to impress upon its readers, especially nurses, the need of a thorough technical preparation for this useful branch of women's work. Comparatively few women have as yet roused themselves to take a wide grasp of the situation—a sort of vague confidence prevails, that inspectors will spring up like wild flowers. Less than half-a-dozen women have as yet got the diploma of the Sanitary Institute, for due study and for passing a subsequent satisfactory examination. It is absurd to fancy that certificates gained merely for attendance at a few popular lectures can prove sufficient preparation for a career of such responsibility as that of an inspector. The Vestry of St. Mary Abbotts, Kensington, is now advertising for "two women inspectors for the better enforcement of the sanitary provisions of the Factory and Workshops Act," and their duties will certainly be many and onerous, for they must live in the district, and devote their whole time to the work. For this devotion they are only offered £60 per annum, a rate of pay which will certainly fail in attracting women of education and special training "between thirty and forty years of age." To board, lodge, and keep herself and to fill an arduous and difficult position for such inadequate remuneration will not attract to any vestry the class of intelligent duly qualified workers, whom the friends of factory girls would like to see appointed. If such good work is at last to be put into the hands of women, everything should be done to attract thoroughly competent ones, and also to induce others to enter at once on a course of preparation which will qualify for the efficient fulfilment of similar duties. Education in sanitation and in personal hygiene are certainly needed in women inspectors, also knowledge of the reasonable needs of their working sisters, and last, but not least, a wide experience of human nature.

### HOSPITALITY TO NURSES.

The Liverpool nurses have been invited to the Town Hall to enjoy two evening parties, given for them by the Lady Mayoress, on September 27th and 28th. These are the first entertainments of the kind which have been given at the Town Hall to nurses, and the arrangements made by Mrs. Robert Holt promise two very pleasant evenings. The kind thoughtfulness which has thus arranged for the repetition of the entertainment is thoroughly appreciated by the invited guests.

### MISTAKEN ECONOMY.

Truth is stranger than fiction, is the experience of most people with large interests and wide sympathies. Some of the strangest truths reported in the newspapers are found in accounts of the meetings of Poor Law guardians. A cold-blooded assertion was made some time since, to the effect that an able-bodied pauper asleep in the ward, was an efficient night attendant for his dying and helpless fellow-creatures. Such a revelation of a living man's honest opinion certainly exceeds any fictitious creation of a novelist's. A guardian at Newtown has recently evolved another curious conviction. He is reported to have complained that "they paid their doctors, and yet were asked to subscribe towards a professional nurse!" He did not consider this a natural sequence; but if the doctors are necessary, surely the nurse must be required for the accurate carrying out of their treatment? The Newtown District Nurse Association has evidently done good work, one poor creature alone having received 170 visits. No one at the board meeting denied the value of the nurse's services, and attention was drawn to the saving to the ratepayers effected by the adequate care which a large number of sick poor had received from her at their own homes. Although the benefits conferred by the Association appear unquestionable, yet the suggested subscription of £5 per annum was strongly opposed, and eventually negatived. Some of the guardians warmly advocated the claims of the Association, but they were overruled by those who held firmly to their scheme of small and illogical economy.

### WORK IN SCOTLAND.

Twelve months ago a Queen's Nurse was supplied to the Forfar District Nursing Association from the Central Home in Edinburgh, and the first annual report, just issued, is a most satisfactory one. The untiring energy of Miss Foulkes, the nurse, and her incessant efforts to alleviate the sufferings of patients brought to her notice, are cordially appreciated by her committee. She has sometimes remained with the sick during the night, when the need for trained nursing has been urgent, and the Forfar Association may be congratulated on the acquisition of so valuable a worker.

### SCIENTIFIC COOKING.

There is one branch of education, and a very important one it is too, which has been sadly neglected in the general education of English girls. There are notable exceptions to the ordinary ignorance, but these are unfortunately few. Every young woman, whether or not she is a nurse, ought to possess a certain amount of knowledge of scientific cooking, not that "fancy work" which deals only with the composition of dainty dishes, but the plain facts treated of by Miss Boland, the accomplished instructor in cooking at the Johns Hopkins Training School for Nurses. She considers that scientific cooking includes a knowledge of the chemical composition of food materials, the methods of preparing food and of preserving it, whether cooked or uncooked, and finally "a knowledge of the law of health, that it may be possible in some measure to determine what constituents and what eatables afford proper material for the maintenance of the body, and under what circumstances of occupation, exercise, and living in general they are most completely utilised."

### OBVIOUS DANGERS.

Nearly everybody buys milk in England. Some folks are obliged to content themselves with a ha'porth fetched in a spoutless jug, or a cracked cup, whilst others have their quarts left for them regularly. All purchasers can tell, with fair accuracy, what becomes of milk, but few know from whence it is procured. "Milk comes from the cow," say the children's reading books, but unfortunately it has to pass through several stages before it reaches the consumer, and its progress is fraught with much danger if the dairy is situated in a poor neighbourhood. The uncovered milk pans are indeed happy hunting-grounds for stray germs, and the milk cans themselves are productive of mischief. Large dairies seldom leave much to be desired in the admirable precautions taken to preserve the milk pure, but the establishments in close and crowded streets lack necessary accommodation, and hence the milk cans are washed on dirty pavements and drained in dirtier gutters. The rinsing being done with most inadequate quantities of water, the condition of cleanliness eventually attained is nominal only. "Where do you get your milk?" might advantageously be demanded by district and private nurses whenever it becomes their duty to administer this most valuable nourishment

### SHORT ITEMS.

The Stonehouse Board of Guardians have appointed Miss Fanny Russell nurse at the Workhouse at a salary of £20.—The Amersham Board recently decided to offer £25 for a "skilled," who need not necessarily be a "a trained," nurse!—The Southmolton Nurse Association has taken the wise step of deciding to engage an additional nurse to attend infectious cases, under medical superintendence.—The Guardians of the City of London Union at Bow offer £50, with board, lodging. and beer money, for a cook for their infirmary, —A committee has been appointed to confer with the medical staff and the present nurses at the North Dublin Union regarding the night duty there—The Guardians at Scarborough have decided to have a certificated head nurse, over thirty years of age. They offer £25 per annum and eight shillings a week in lieu of rations.—At Warminster Mrs. Gaymore has been engaged as nurse for the Infectious Hospital. She is sixty years of age, and has lived for twenty years in India.—*Nursing Notes* for October contains some good medical and nursing articles, besides an interesting communication from the Sub-Committee of old-workers of the London Hospital, and a quotation from a letter written by the Secretary of H.R.H. the Duke of Cambridge.

### Insanity Among Coloured Races.

(BY OUR GRAHAMSTOWN CORRESPONDENT.)

### AFRICAN ASYLUMS.

ONE of the first things that strikes an English nurse as unfamiliar in a South African asylum, is the division of the building into two parts, one reserved for the European patients, the other for the coloured people. This separation is carried out in every department of the daily routine, the native patients having very little association with the Europeans. It is a necessary and desirable arrangement for many reasons, one of which is, that the undeniable antagonism which everywhere exists between the white and the coloured races is no less apparent in the asylum wards than in the outside world.

It has always appeared to me very difficult to understand the existence of insanity among these coloured races. Their lives are so simple that they are free from the cares and worries incident to civilised life; their wants are so few that poverty is unknown among them, and at the best of times they exercise so little self-control, that the border line between sanity and insanity is still harder to define in their case than in that of civilised peoples. To a stranger, especially, who does not understand their language, there is little or no perceptible difference between a sane and an insane native; yet that some difference does exist is evident from the superstitious terror with which natives outside the asylum regard all the patients, both white and coloured, within its walls. In determining the sanity or insanity of an uncivilised native, it is necessary to recognize the fact that many things which would be undoubted symptoms of insanity in a civilized person, in his case are the natural acts of his daily life. If a white person were to strip off all his clothes because it was a hot day; to dance and sing (or rather yell) all night long when the moon was shining brightly; to eat his food with his fingers, and occasionally help himself off his neighbour's plate; to appropriate any article of clothing or other portable property that he might happen to find lying about; to collect any quantity of rags, pieces of glass, and rubbish of all descriptions, and carry it about continually in a large bag; to gesticulate wildly whenever he spoke; and to come to blows directly he was seriously annoyed, he would, with some justice, be considered insane, and his friends would probably have no difficulty in obtaining certificates to that effect. But all these things a native may do, and will do, every day of his life, and still retain the reputation of being perfectly sane, Unless, therefore, there is clear evidence of delusions and hallucinations, incoherence of speech and ideas, or actual imbecility, it is impossible to say with certainty that an uncivilized native is insane.

This leads us naturally to conclude that the causes of insanity must be fewer, and simpler in their action, among uncivilised than among civilized races. Heredity can scarcely be taken into account, as it is very seldom that anything can be known of the family history of a native patient, even his own name and age being frequently a matter of grave doubt. Drink is, of course, a predominant cause, and so is habitual smoking, especially of a plant named Dagga, which the natives are very fond of using for this purpose, and which seems to have a most confusing and exciting effect on the brain. Maddened with brandy and dagga, the natives outside the asylum will indulge in fights and orgies quite worthy of those inside it, and at such a time the two would be by no means distinguishable.

Another cause which, I venture to think, is widely operative, is the too great pressure of an artificial civilisation, and a European education, which have been forced on untamed savages in the course of two or three generations. The Kaffirs (to take an instance), are very quick and intelligent, and will acquire some kinds of knowledge with surprising rapidity. A Kaffir can take his degree now; but is it reasonable to suppose that a native, whose grandfather was a "wild man of the woods," and whose grandmother, perhaps, was of the lower race of Hottentots, can have a brain sufficiently developed to bear the same strain as the brain of the descendant of hundreds of civilised and educated ancestors?

These semi-civilised natives (for the civilisation is only skin deep at present) are much more troublesome to manage, when insane, than the raw savages. The women develop quite a civilised form of hysteria, as in the case of one girl of seventeen, who had periodical attacks of hysterical mania, which she would bring on, as such patients will do, by putting herself into a violent temper, and gradually working herself up to an attack. Indeed, she was the greatest adept at "putting on" that ever dwelt within asylum walls, and had, at the same time, as thoroughly savage a nature, when the varnish was rubbed off, as ever came out of the African Bush.

We are forced to acknowledge, therefore, that the chief causes of insanity among the coloured races, seem to be the strain of a too rapid civilisation, and the abuses that accompany all civilisation.

Epilepsy, as far as my personal experience goes, is apparently very rare amongst natives. The only two cases that have come under my own observation were both women of mixed race, the one having Indian blood in her veins, and the other probably Dutch. I have never myself known a pure Kaffir to be an epileptic. It would be interesting to know if epileptic insanity is also a development of civilisation, or whether it results in any way from the mixture of races.

Native patients are, on the whole, more habitually violent than white patients and more objectionable in their habits, which is only what one would naturally expect. At the same time they are more manageable from the fact that they are accustomed to be always subordinate to the white man. They collect rubbish to an incredible extent, and carry most of it about their persons; and they are, as a rule, splendid workers, the women having the strength of men, and doing nearly all the hard work of the institution, such as scrubbing, laundry-work, &c. Their great muscular strength makes it harder to restrain them when they are violent, and occasions greater risk to those who have charge of them. Being at the same time less sensitive, they require to be more sharply and firmly controlled than European patients. On the other hand they are more faithful in their affections than is common amongst lunatics; as anyone returning to them after absence will soon discover, from the enthusiastic warmth of the greetings he will receive from every patient who has any sense or memory at all. And no one could fail to notice the striking difference between their reception and that of the European patients, who, for the most part, seem hardly aware that one has been absent at all.

My own experience is that native patients appeal very strongly to one's sympathies and affections. They are, for the most part, simple and childish in character—cut out of the raw material, so to speak—yet they are both interesting and attractive; and they are suffering under a curse which, I cannot help feeling, we have, to a great extent, brought upon them.

### A Hint for Ward Nurses.

THE surplus bedding plants will be distributed to the public on application to the superintendent at the various parks on the undermentioned dates: Battersea Park, October 24th; Myatt's Fields, October 21st; Ravenscourt Park, October 21st; Royal Victoria Gardens, North Woolwich, October 14th; Southwark Park, October 27th; Victoria Embankment Gardens, October 13th; Waterlow Park, October 18th.

### Nursing in Paris.

(By a Wandering Nurse.)

### II.—INSIDE THE HOTEL DIEU.

The lounging students pulled themselves together at the approach of the Chief. They were of all ages and from all countries—dark, fair, tall and short (but chiefly short!); all earnestly desiring to hear and to see the physician. He was well worth looking at too, for his thick grey hair, worn rather long, covered a fine shaped head, and the rugged features and quick eyes were peculiarly impressive. The Sister first claimed his attention, presenting a number of forms, to each of which he rapidly appended a signature, talking fast all the while. When this was done he suddenly turned to the "resident" in attendance, and thence ensued an amusing and curious scene. Some action of the junior's had evidently displeased his senior; apparently a small breach of etiquette regarding the transfer of a patient to another department, was the cause of offence. Whether the matter were small or great, it resulted in angry impatience on one side, met by explanations and arguments on the other.

For a Chief to be displeased with his sub. is by no means an unknown circumstance, but for the fact to be made patent to 28 or 30 patients and nearly twice as many students and attendants, is unedifying and unusual. However, when the storm had passed, the clinical lecture began, and with rapid eloquence, the history and symptoms of each patient were recounted by the doctor, and his audience was alternately instructed, advised, and questioned. Hearing a slight bustle, I glanced round to see a white-haired old man on a stretcher, deposited in the middle of the ward. The lecturer's voice still went on, so I was content to listen now, without watching his descriptive gestures, and let my eyes wander to the new group of persons. Two attendants wearing long linen blouses or shirts, and very loose white linen trousers over their dark ones, began to prepare a bed for the patient. They put on clean sheets, and made up the bed very deftly, and then the sister approached them. She was a little woman, in a voluminous "religious" garb; a loose coarse white linen frock with "Bishop's" sleeves, covering her thick black robe, except for the space of a few inches above the hem. Over this primitive linen garment a coarse bib-less apron was worn, and rosaries, instruments and keys were suspended by various cords from her girdle. She proceeded to turn down the upper bed clothes, giving a little pat to the pillow, and then the attendants lifted the old man quickly and laid him down very gently. The whole performance was exceedingly swift, the male nurses being not only rapid but quiet, and their shoes seemed absolutely noiseless.

The old man was accompanied by the prettiest old woman imaginable. A frail little creature, with china blue eyes and hair as white as her charming starched cap, and a certain little air of dignity pervading her neat figure and flushed face as she stood by the new patient's pillow. He was her brother, she said, when questioned by the doctor, and to all inquiries she gave clear and intelligent answers, keeping her place through the long lecture which followed. "Anticipating judgment day," flashed into my mind as I heard the questions put in rapid succession. Surely, no "confessional" could exceed the closeness of these investigations. However instructive to students, I felt it was hardly in my line, so I turned aside out of the crowd of men and looked around the ward again. Plenty of windows, but all of them shut, and the atmosphere close and heavy in this crowded medical ward. One student apparently felt the heat was becoming stifling, and he ventured to open a distant window and put his head through for a moment, but he soon closed it, very softly, like a boy conscious of breaking the rules.

A wooden tray on three large wheels was noiselessly pushed into the ward by an attendant, and the Sister standing by it measured out the daily allowance of light wine for each patient, then a young girl, in a similar habit distributed slices of white bread cut from a very long roll. One of the attendants was occupying himself with a hypodermic syringe, which he cleaned with great precision.

Suddenly the lecturer raised his voice and called out for the Sister. She responded briefly in a clear, high tone, and remained on the outskirts of the closely-packed group of men, whilst a prescription was dictated to her, it was a sleeping draught, to be given at bedtime. The diet for the new, and the changes in the treatment of the old, patients were also communicated to her in the same informal manner. In the meantime a student added these orders to the notes which he had already jotted down, on a form provided for the purpose, The beds were very close together in this ward, and there were two or three on trestles down the middle. These were certainly ill-adapted for sick folks, and obstructed the thoroughfare. They were also occasionally sat upon by the students, which was trying for the invalids. The mattresses were all good, and the linen white, though coarse, an extra sheet serving for coverlet. The bedsteads were ugly and rather clumsy, with an iron shelf attached, in the exact position to secure for a patient attempting to sit up, a nasty blow on his head. On this shelf various properties of the patients were placed, and unpleasantly conspicuous were the vessels for receiving, measuring, and saving urine. There were bed curtains in some of the medical wards, but not in all. They appeared to be altogether abolished on the surgical side of the hospital, although the iron framework for them still remains intact.

Instead of iron or tin receptacles, bright copper vessels contained the cooked food sent up from the general kitchen. These were kept hot on stoves in the little kitchens adjoining the wards, pending the distribution of diets. The stews and vegetables all looked very good, and the white crockery was placed in readiness for conveyance on the light three-wheeled trolleys or tables, which plied to and fro. Piles of snowy linen also travelled on similar vehicles, to the polished wooden presses in the store-rooms.

The surgical wards seemed much less crowded and better ventilated than the medical, and the long windows between each bed were, most of them, wide open, admitting the glorious sunshine and fresh air.

There appeared great neatness and infinite care bestowed on the removal and replacement of dressings. There was no disorder at any time, and the floors were kept as spotless as the tables, being protected by a sheet of mackintosh whenever there existed a possibility of a catastrophe. Accustomed to the flowers and plants which adorn our English hospitals, I was surprised to find them totally absent from the French one. There was no visible sign that the care given to the patients within, was supplemented by gifts such as these, from without.

No wonder our neighbours across the Channel disdain our homely family fruit pie, when the dish which contains it is the facsimile of those which they use for the dressings! The white china jars which are popular in some hospitals for moist dressings are replaced at Hotel Dieu by large, clear, glass vessels, whose lids not only fit the top, but have a deep overhanging rim of glass, which overlaps the jar, and renders it well nigh impossible for germs or dust to penetrate.

Smoking is only forbidden to the patients during the doctor's rounds. At all other times the inevitable cigarette is permitted unless refused to individual cases by the doctor.

Lay nurses wear no uniform but only a large white apron over an ordinary lady's dress, and, of course, a stranger in a single visit is quite incompetent to judge of the prevalent system of nursing. The cleanliness of the whole establishment left nothing to be desired and the polished floors were in excellent condition.

At night three of the sisters are responsible for the supervision of the hospital.

Time only permitted of my visiting portions of the building in which I was allowed such unrestricted wanderings, but I shall always remember with pleasure my morning at the Hotel Dieu.

### Modern Nurses.

(By an Experienced Matron.)

So much has been said about nurses lately that it would be, perhaps, a relief to hear no more of the subject, if silence would not seem to "give consent" to the opinions of "unprofessional" people, who take but a limited view of the subject on which they write. Reading their remarks reminds one of a well-known doctor's description of "smell fungus"—a term, he says, "applied sometimes to persons who go about the world sniffing for grievances and trying to set everybody right." Those in responsible positions who know what nurses really are—and how few, comparatively, are the failures in their ranks—read with indignation the misleading statements on the deterioration of nurses. But the movement is still forward; the tide has not turned. At no time in the history of hospitals has such good work been done as at present by the army of intelligent, well-trained nurses, in this country and elsewhere.

They have facilities for learning their profession thoroughly, which were unknown to nurses a generation ago.

They are well prepared and instructed in their work by the excellent courses of lectures, which in nearly all training schools are systematically and willingly given by the medical staff, who recognise skilled nursing as an auxiliary in their work. If those who write so lightly and glibly of the shortcomings of the few nurses who have come within their ken would consider what is meant by "modern nursing" they might realise the extent of the work that is being done by thousands of women who, without making any pretence of being superhuman or wonderful, are content "to spend and be spent," giving the best powers of body and mind to their work.

For one who lifts up her voice and complains, there are ninety and nine quietly stationed at their posts, who care only to use to the utmost the vast opportunities, and high privileges, that belong to their calling. It is well to remember what is being done by the "trained nurse," and how she is found in all countries, from the highly equipped hospitals in Europe to the makeshift huts of Central Africa and the far regions of Corea, nursing the lepers in the cold climate of Scandinavia, under the tropical sky of the Sandwich Islands, or in the hot sandy region of Robben Island. Nursing in temporary cholera huts amid the excitement of an epidemic, and tending the sick and dying paupers in the monotonous routine of the poor-law infirmary ward.

There is no better service rendered to the poor in this country than by the devoted district nurse in the crowded alleys of our great cities, or in country parishes, where she tramps through lonely lanes to outlying hamlets to bring help to the patient. These are some of the ways in which thousands of women work day by day, of each of whom it may truly be said, as St. Paul wrote of one who was probably the first Deaconess in the Christian Church, "She hath been a succourer of many."

And all this record of noble work is ignored, and the mention of the modern nurse is met with a sneer because a few—unworthy of their calling—are seen walking in Regent Street with fringes and high-heeled boots!

There are some nurses who apparently have no loftier ideal than to make themselves conspicuous, but these are the tares among the wheat, and only those who wilfully shut their eyes, can be blind to the marvellous harvest that from the smallest beginning—a seed sown within the memory of man—has grown up and spread out branches far and wide.

### Nursing in South Africa.

(By a Practical Nurse.)

### A COTTAGE HOSPITAL.

The Victoria Cottage Hospital at Wynberg—an important suburb eight miles out of Cape Town—was established 1887. It stands in a high and healthy position with splendid views of the mountains, and the wards are bright and cheery. Just now painting and cleaning are going on, also the building of two more wards, bath room, &c., which will all be great improvements, as the white and coloured patients, men and women, will then be entirely separated. The building is all on one floor, there are four wards, two with four beds in each, one for men and one for women, and two private wards with two beds in each; and there are two cots which can be moved about as occasion requires.

There are five doctors connected with the hospital, and these take it in turn to be "medical superintendent" for the week.

Every patient has to have a recommendation for admission except in cases of accident, and there is a regulated scale of charges to suit most cases, and there are also three or four free beds. The small wards if used for only one patient are 7s. 6d. a day. A nurse recovering from typhoid contracted whilst nursing a patient was in one of these wards, and it looked cosy and comfortable. Labourers pay 9d. a day, and domestic servants 1s. or 1s. 6d., according as they are in or out of service, &c. The operating theatre is small but very convenient.

The present Matron was trained at University College Hospital, and also at Edinburgh Children's Hospital, and she has only been out a few months; she has two nurses, one for day and one for night duty; one of these can speak Dutch, which is very convenient as they constantly have patients who cannot speak English, and it must be very trying both for patient and nurse not to be able to understand each other's language. The bed-rooms are very small, but the nurses have a pleasant sitting-room, and the matron's when free from the confusion of being painted, &c., must look very home-like and comfortable. The matron is under the authority of the Sister at the Somerset Hospital who comes out once a week to see after things.

The hospital receives an annual grant from the Government, and the rest of the cost of its maintenance is met by patrons, life-governors, church subscriptions, and sums collected by ladies.

It must be easy to keep the wards pretty with such profusion of lovely flowers; roses, violets, and heliotrope grow in abundance in all gardens, there are lovely heaths of all kinds and innumerable dainty mountain flowers. Imagine great clumps of pure white arum lillies growing everywhere wild, with their thick glossy leaves.

As I write it is two o'clock in the morning, and I am sitting in a comfortably furnished room with a bright fire of wood and fir cones, watching beside a very sad case I am helping to nurse. The patient is slowly and painfully dying with cancer of the liver. There is little to be done except the administration of opiates for relieving the pain, and small quantities of fluid nourishment. I cannot say enough of the kindness that I receive from everyone, not only from each member of the household, but from all their friends.

### Where to Go.

Free Lectures at Gresham College.—Dr. E. Symes Thompson will deliver four lectures on "Physic," in the theatre of Gresham College, E.C., commencing at six p.m., on Tuesday, Wednesday, Thursday, and Friday, October 10th, 11th, 12th, and 13th.

### District Nursing.

(By Another District Superintendent.)

As a District Superintendent I am very much interested in the two articles which have appeared as a sequence of Mrs. Dacre Craven's account of district work. Though I had not the privilege of being actually trained by Miss Florence Lees, I began district nursing at the time when she still took an active part in the supervision and inspection of the work, and so may claim to have been fully under the influence of her teaching. I cordially endorse the opinion of "An Association Superintendent" that "Miss Florence Lees was most practical and wise in her organisation of the work in London," and also the necessity of her maxim of "thoroughness, even in the smallest things."

But I also agree with a "District Nurse" that it is not wise "that a trained nurse for the poor should be looked upon as a 'parish charwoman.'" Only those who, like myself, have had experience in beginning new branches in different parts of London, or have helped to start the work in the country, can understand how prevalent is this mistaken idea.

It is but the survival outside the hospitals of what was formerly the rule within them, that whatever work was to be done must be performed by the nurses. If, by the universal consent of the authorities, this work of scouring floors and polishing grates has been delegated to wardmaids and scrubbers, why must trained nurses scrub and sweep the patients' rooms in the district?

If the "ordinary uneducated woman" can be trained and trusted to perform these duties for the severe cases of illness in the wards, why may she not do the same for her own friends and relations in their homes, under the direction of the nurse?

In all other professions and trades it is recognised to be false economy to employ skilled labour for work which does not require it. Why should district nursing be the exception?

Those who employ a district nurse require a certain standard of work from her, and this she cannot give if mind and body are alike wearied in efforts which not only unfit her for the work she alone can do, but deprive another of an opportunity of employment and improvement.

District nursing, like everything else, must "march with the times." Influences are now being brought to bear upon the people, that were only in their infancy when this work began. The steady repression of lazy thriftlessness, with its accompaniments of dirt and squalor, the efforts to make men and women self-helpful, self-respecting members of society, must be aided, not retarded, by the district nurse.

This very education of the friends to take their share of the nursing work in the right way, is part of the nurse's mission. It is only carrying out Miss Nightingale's teaching, that a good head will train the subordinates to perform their own duties in the best possible way—not try and do everyone's work for them.

Besides, to look at the question very practically, sweeping and scrubbing do not come naturally to any nurse or superintendent. They require training to do them really well; it is not so easy as it looks to wield the long broom, polish a grate, or scour a floor, as I know by sad experience. Bits will stick to the carpet, the blacklead will not "shine," and "tide marks" appear on the vigorously scoured floor when it has dried.

I doubt the soothing influence of the nurse's well-meant efforts on the professional charwoman, for instance, though politeness and gratitude for the intended kindness may prevent her showing her real feelings.

The old saying, "Cobbler, stick to your last," seems applicable in this case.

Another point is that evidently the "Association Superintendent" can spare two nurses to each case—one "to help" the other. In nearly every instance, however, the nurse is single-handed, therefore double the time and strength would be expended.

I have gathered from my own experience, and by trying both methods many times, that, except under exceptional circumstances, for which a well-trained district nurse is always prepared, more abiding influence and more thorough lessons in order and cleanliness are produced by making the relations and friends do as much of the actual work of nursing as they can. We want to help, not to pauperise our patients, and the truest way is to teach them to help themselves.

No district nurse worthy of her calling wishes to pose before her patients as being perfection. She goes amongst them to help and to influence, not to patronise, frankly asking for their help in return.

As to questions about spoiling her hands, or running up a list of visits as evidences of work done, they are better left unnoticed, put out of sight amongst the far higher and greater interests involved in this most important branch of nursing effort.

### Everybody's Opinion.

[Correspondence on all subjects is invited, but we cannot in any way be responsible for the opinions expressed by our correspondents. No communications can be entertained if the name and address of the correspondent is not given, or unless one side of the paper only be written on.]

### WATFORD DISTRICT COTTAGE HOSPITAL.

Mr. H. Morten Turner, hon. secretary to this hospital, writes: Miss Hillyer's letter published in your issue of 16th inst. contains the following passage: "I entirely attribute my having in this instance fallen a victim to infection to the fact that for forty-three hours after the operation (tracheotomy) I was unable to leave my patient for a moment, consequently what food I had was eaten in the ward, proper rest and fresh air being, of course, out of the question. The hospital was nursed by myself and a nurse who had only had the training which the limited opportunities of a cottage hospital could give her, and she had never seen a case of tracheotomy. It would, therefore, have been culpable on my part to have allowed her to nurse it even if she had not to take charge of the other patients in the hospital. I could not leave critical cases to untrained care, and was therefore sometimes obliged to nurse a patient day and night for a week together without any proper rest." Commenting upon this statement you not unreasonably observe that the fact of any person having to remain in constant attendance on a tracheotomy case for forty-three hours is well nigh incomprehensible in a place near London, where the temporary aid of a trained nurse could be promptly secured. On this comment permit me to remark that Miss Hillyer had express authority to telegraph to London for a trained nurse at any moment; and exercised this power on various occasions, so that her inability to leave the case, and her obligations to nurse day and night, arose solely from her own deep personal interest in the cases committed to her charge, and should not be attributed to neglect or thoughtlessness on the part of the committee or the medical staff. I am sure Miss Hillyer would very much regret the construction which has been placed upon her letter. The nurse who had never seen a case of tracheotomy had, however, had the advantage of being under Miss Hillyer for nearly four years, and during the illness of the latter satisfied the medical staff that she had made very good use of her opportunities. In conclusion, I should add that the hospital is strictly a cottage hospital, containing only nine beds, and that the admission of infectious cases is absolutely prohibited. On one occasion a patient admitted for another complaint developed scarlet fever, and one other patient contracted the same disease. The case of membraneous croup, to which Miss Hillyer alludes, was admitted by her under circumstances which entirely justified her in that step, but necessarily without the previous sanction of the medical staff or committee.

### Notes from Philadelphia.

(By our own Correspondent.)

### NURSING AT THE METHODIST EPISCOPAL HOSPITAL.

The Methodist Episcopal Hospital, South Broad Street, was formally opened for the admission of patients April 21st, 1892, A. Rittenhouse, D.D., being appointed as Superintendent, and Miss Linda Richards as Directress of Nurses.

Miss Richards has a wide reputation for the successful organization of training schools for nurses, and has had long and varied experience in the work. In 1873 and 1874 she reorganized the training connected with the Massachusetts General Hospital in Boston, Mass., and she left there to take a course of instruction in St. Thomas's Hospital, London. After visiting many, and remaining some time in one of the hospitals in Edinburgh, she returned to America and formed a training school in the Boston City Hospital. After some eight or ten years Miss Richards resigned, and went to Japan to establish a training school for native women. She stayed there for four years, and was desirous of continuing longer at that interesting work, but her health necessitated her returning to her native country. When her health improved she accepted the position of Superintendent of the Visiting Nurse Association of Philadelphia, and by her experience and executive ability greatly aided the managers in improving and extending its efficiency and usefulness. So highly were her services appreciated that the managers had her appointed on the executive board, of which she continued a member as long as she remained in the City.

She spent some time in the insane department of the Pennsylvania Hospital, but finding them not quite ready to establish a training school there she accepted the position of Directress of Nurses from the managers of the M. E. H., and began the work of organizing the school on the formal opening of the hospital. The climate of Philadelphia being a little trying, and the duties more arduous than her health could stand, Miss Richards resigned this post, and accepted that of Superintendent of the New England Hospital for Women and Children in Boston, Mass.

In January, 1893, one of Miss Richards' former pupils, Miss Jessie J. Glen, a graduate of the Boston City Hospital, was elected to succeed her as Directress of the Philadelphia Training School for Nursess. Every facility for thorough training in theoretical and practical nursing is offered by the hospital. Lectures are given by the medical staff and the pupils are required to take notes. Class recitations are organized by the directress and examinations take place at stated periods. Lessons in invalid cookery are given. During their second year the pupils are sent out to do private nursing and the hospital receives all the payments made for their services. Diplomas are granted at the end of two years, and pupils are eligible between the ages of 23 and 35 years, of every Protestant denomination. The school consists of one graduate, six senior, eleven junior, and two special nurses, the latter from the Deaconess's Home.

The graduate, Miss Tetsu Ito Lan, is a Japanese and received her training in Miss Richards' native school. She came to this country to study our methods more fully, and after a few months in the M.E.H. she expects to go to the Boston Lying-in Hospital for a time, and afterwards to visit and study the application of electricity, massage, and baths as practised in some of our sanatoriums. She will then return home and give to her countrywomen the benefit of her wider experience. She is doing all this at her own expense from pure philanthropic motives.

One of the senior nurses, Miss Elizabeth Wind, is an Indian girl from the school at Carlile, Pa., who, after her graduation, expects to return to her tribe (the Wyondottes) to practice and teach her profession.

### For Reading to the Sick.

### THE CROSS.

We have a tradition brought down to us about the finding of the real cross on which our Lord Jesus Christ died, and it is one from which we may learn some useful lessons. For two or three hundred years after our Lord's ascension the cross was supposed to have been destroyed, but at the end of that time the Empress Helena, the widow of a Roman emperor, a devout and holy Christian woman, determined to visit Jerusalem, and especially the scene of the crucifixion, in hopes of finding the wood which has proved to all believers the "Tree of Life." She took a great number of persons in her train, who with other workmen living on the spot dug all over the place in which our Saviour had made his tremendous sacrifice, and they were successful in their search. Three crosses were found buried close together deep in the earth of Golgotha, one of which they recognised as the "true cross." With joy the Empress divided it into three portions, one of which she sent to Rome, one to Constantinople, and the largest part she enclosed in a shrine which was deposited in the Church of the Holy Sepulchre at Jerusalem, built for the purpose. There it remained for several hundred years more, when the king of the neighbouring country, Persia, besieged the Holy City and carried away its treasures. The one he valued most, however, was the portion of the true cross, for he thought that as the Son of God had died on it it must have great virtues and would protect him from all harm. But the king remained a heathen till the day of his death, and in consequence we never hear that the possession of the cross was any advantage to him. In a couple of hundred years another Roman emperor, Heraclius, took the precious relic away from the Persians and restored it to Jerusalem again.

We may ask how it was that the cross brought no blessing to the King of Persia? Simply because our Lord Jesus Christ was not with it. We Christians call an affliction, an illness, a trouble, in short anything which annoys us "our cross," and we are apt to think with the heathen prince that those crosses themselves will keep off the punishment of our sins. Unfortunately they sometimes have a contrary effect and instead of softening our hearts, render them irritable and selfish, so our last state is worse than our first. And this is so because we too have not Jesus Christ with His cross. He is always ready to come with it, for He is so loving that He would be with us at all times if we would let Him, especially when we are in trouble and are bearing our cross of suffering, only we will not take the burden from Him. We like to have the choosing of our cross ourselves, whereas He only knows what we can carry. If we will put our hearts and shoulders at His disposal we shall find His burden light, and our cross turned to flowers in the bearing of it. If our trials are as the nails and spear, the vinegar and the gall which our dear Saviour suffered when extended on the lofty tree, all will turn to sweetness if with the cross we enshrine in our hearts that "sweetest Weight" which hung on it "for this world's ransoming."

### Minor Appointments.

Fountain Fever Hospital.—Miss May L. C. Browne has been appointed a charge nurse at the Fountain Hospital. She was trained at the New Infirmary, Birmingham, was afterwards out-patient and theatre nurse at the Eye Hospital in the same town, and charge nurse at the Ipswich Fever Hospital.

Miss Gertrude L. Clayton has also been appointed charge nurse at the Fountain Fever Hospital. She was trained at the Jaffary Hospital, Birmingham, and was for five and a-half years charge nurse at the Birmingham and Midland Eye Hospital. Afterwards Miss Clayton held the post of sister-in-charge of the Borough Fever Hospital at Ipswich. Many good wishes will follow both nurses to their new work, in which we cordially desire for them every success.

### The Muses' Looking Glass.

### PITFALLS IN PAMPHLETS.

A log of wood may wreck a train, a small match may lead to a ghastly explosion, and a simple health tract, a little unpretentious pamphlet, may prove a deadly weapon in unaccustomed hands. "A little knowledge" is not necessarily "dangerous," in spite of the old proverb; but it becomes so when it leads people, knowing nothing of drugs or of physiology, to write pages of advice for distribution amongst their uneducated neighbours. These tiny books often attract the unwary, and are conscientiously perused by the innocent searchers after knowledge.

Charles Kingsley's admirable and wholesome tract, entitled "The Two Breaths," is an ideal production, and his "Massacre of the Innocents" is equally forcible and true; but few pamphlets follow his lines. Least of all such books as one called "Six Lectures on Cottage Nursing" by a "Quiet Friend."* The writer is evidently untrained to judge of practical points, or she would hardly recommend cotton as desirable padding for circular pillows. However, this and sundry other bits of advice, are small matters compared with the serious blunder of dictating treatment for diseases. The simpler English names for these should always be used in lectures to cottagers, and not such terms as pneumonia. Certainly a jacket linseed poultice as treatment for the latter should be left to the doctor to order. Instruction in poultice making and the preparation of plasters and fomentations is excellent work if practically carried out, and the "Quiet Friend" has certainly gone fully into these matters, but even here she ventures on prescriptions, and actually recommends the sprinkling of laudanum on fomentations, without any limit as to the quantity of the poison. Again, compound opium liniment is calmly advised without mentioning the necessity for a doctor's authority for its employment.

For burns and scalds, which are assuredly amongst the most serious of calamities, free advice as to dressings is given, but no hint of the first danger to be encountered and conquered, viz., the symptoms of "shock." Especially in the case of children this should be attended to first. Fussing about after dressings for wounds, seems to come naturally to everybody, but an immediate endeavour to counteract the patient's terror and subsequent collapse, can always be advantageously insisted upon, to untrained as well as to professional hearers. We may regret the omission of practical points in pamphlets designed to be helpful, but a much stronger sentiment is aroused on finding the diagnosis of scarlet fever disposed of in two lines, and evidently considered a matter within everyone's comprehension. Medical men who make a special study of fevers will hardly endorse such a presumption. For kidney disease, as a sequence to scarlatina, active treatment is laid down, quite independently of any medical advice, which is somewhat alarming teaching. Leeches are spoken of as successful treatment for glandular swellings in the early stage, but we do not hear who is to order them.

"No medicine to be given while the rash is out" is a decision we have hitherto imagined lay entirely within the province of a doctor, but the heroic "Quiet Friend" disdains such ordinary proceedings. She decides for herself that when peeling begins "baths must be given at once." What is left for the doctor to do? Perhaps he is to write the death certificates when he is called in too late for aught else.

"Measles" is described as "an infectious but not a dangerous disease." How is it then that hundreds of little children are its annual victims? Most authorities concur in avering that it is just this very failure to acknowledge the danger which causes so many victims to measles to swell the rôle of the martyrs to preventable diseases. "Condy's fluid may be kept with advantage in one or two jars in the room," is another puzzling sentence, and the critic fails to see where the "advantage" lies, especially if the said jars happen to be carefully corked.

Diphtheria is diagnosed and prescribed for in the Cottage Lectures, several remedies being advised for application "by means of a camel's hair brush to the surface of the throat." If this terrible disease often receives such extremely casual treatment, there may be some foundation for the writer's remark, "The difficulty of attending to such severe symptoms in children undoubtedly causes the number of deaths during the first five years of childhood." What can be said as to the accuracy of the statement that in nursing diphtheria "those attending can do so without fear?" Without selfish fears by all means, but certainly not without danger to themselves if they have no further directions given them than to "gargle occasionally with Condy's fluid and to take some nourishment." Is the fluid to be used undiluted, and where is the nourishment to be taken, inside or outside the sick room? This essential point is left unexplained, and not a word of warning is given as to the real source of danger and the need for every precaution.

Perhaps the greatest danger in this manual is advice regarding the administration of laudanum. There is no hint given as to the need for a doctor's authority in this important matter. Injections are to have ten drops of laudanum added occasionally, and in another paragraph fifteen to twenty drops is the quantity advised. But even this limit is sometimes set aside, and what will the cottager make of this sentence, "Morphia for the relief of cancer and other diseases of the rectum."

In the remarks on typhoid we meet with a good deal of interjectional advice, such as "stimulants, as a rule, should be increased during the second week." As there is no suggestion of the doctor's permission being obtained, we can draw a pretty accurate mental picture of the way in which these vague directions may be observed.

Some relic of the dark ages appears to have crept into this production of 1892! For we are told "cob-web rolled into a ball will stop bleeding." Where are our boasted antiseptics and our modern knowledge of hygiene when this is still the teaching meted out to our long-suffering English poor?

In sunstroke we read that "croton oil is generally given at once." This is usually dropped on to sugar (no quantity specified) "and placed far back on the tongue." For neuralgia, "subcutaneous injections of ether" are amongst the remedies for home use. Cramp in the stomach is destined to receive the heroic, if somewhat uncertain, treatment of "brandy and opium, with mustard plasters to the legs." A cough mixture is prescribed containing laudanum, and this favourite drug is also recommended for colic. Altogether the "mothers and wives of working men," for whom this original and dangerous little pamphlet is designed, will have an opportunity of picking up hints on drugging, on the diagnosis of infectious diseases, &c., which must sooner or later land them in the doctor's, and possibly in the coroner's hands.

* "Six Lectures on Cottage Nursing." By a "Quiet Friend." (Printed by Messrs. Wilson and Phillips, Hereford, price 4d.)

### Notes and Queries.

### SPECIAL NOTICE.

The contents of the Editor's Letter-box have now reached such unwieldy proportions that it has become necessary to establish a hard and fast rule regarding Answers to Correspondents. In future, all questions requiring replies will continue to be answered in this column without any fee. If an answer is required by letter, a fee of half-a-crown must be enclosed with the note containing the enquiry. We are always pleased to help our numerous correspondents to the fullest extent, and we can trust them to sympathise in the overwhelming amount of writing which makes the new rules a necessity. Every communication must be accompanied by the writer's name and address, otherwise it will receive no attention.

### Queries.

(201) *Inspection.*—What prospects of making a livelihood are held out for women as sanitary inspectors?—*Sanitation*

(202) *Examinations.*—Kindly tell me the rules for the examination questions.—*Enquirer.*

### Answers.

(201) *Inspector (Sanitation).*—An explanatory paragraph on this subject appears in another column of the Nursing Supplement this week.

(202) *Examination (Enquirer).*—The rules, which are very simple, accompany each question. The last one appeared in The Hospital of September 16th, page ccxlvii.

### The Book World for Women and Nurses.

[We invite Correspondence, Criticism, Enquiries, and Notes on Books likely to interest Women and Nurses. Address, Editor, The Hospital (Nurses' Book World), 428, Strand, W.C.]

Queen Olga's Evangelismos.

Madame Hélène Lascaris has furnished in *La Nouvelle Revue* a description which cannot fail to be of interest to our readers, of the work initiated and carried out by Queen Olga among the sick in Athens. So recently as 1872, Greece was almost wanting in doctors, and the profession of a nurse was unknown. It was then that certain ladies of high rank in Athens founded an association for the training of sick nurses, which developed later on a nursing school, and recently a hospital. The hospital, or as the Queen prefers it to be called, the Evangelismos, is directed by Madame Syngros. It is composed of three distinct buildings, united by covered passages, with operating-rooms, surgery, mortuary, and offices detached. A hundred and thirty in-patients, and about sixty out-patients are under treatment, and of these about one-fourth contribute payment. There is a magnificent garden for the use of patients who are able to enjoy it, and a pavilion has been erected in it for the special reception of patients recovering after operations for tumour. All nurses admitted to the association sign an engagement (after a year's noviciate and the passing of an examination) to remain for at least six years, and not to marry during this period. A ladies' committee of seven meets once a-week and manages all the affairs of the hospital. One of these ladies in turn takes special charge for the week, and passes the whole morning in visiting the wards, inspecting the rations, and giving "necessary instructions," but in addition to this the ladies' committee pervades the hospital freely at all hours. The Queen herself acts as treasurer and director-in-chief, and takes an untiring interest in the patients, whom she constantly visits. The tender solicitude of the Queen on behalf of all sufferers is extended also to the municipal hospital of the Piræus. She visits it frequently, and has told off one of the nurses (infirmières) of the Evangelismos to work there, and as she has long lived in Russia and speaks several languages she is like a providence to the foreign seamen. The Queen, who has always abstained from favouring her compatriots by conferring on them honourable posts, shows her sympathy by visiting them regularly in illness. They say even that she has had earth sent her from Russia that she may sprinkle it with her own hand on the grave of those Russians who die far from their country. During her late visit to Paris, the Queen consecrated many days to visiting the hospitals, making careful inquiries and taking note of all improvements and of the progress accomplished in each. She pursues simply and without ostentation the task she has imposed on herself, considering herself as much tied by her duty as the nurses by theirs. One thought appears to reign in the hearts of all those who co-operate in the work, the precept inscribed by order of the Queen in different parts of the building, "Love one another."

Woman Free. By Ellis Ethelmer. (Published by the Women's Emancipation Union, Congleton.)

Mrs. Ethelmer's book is about the most courageous work that has yet appeared on behalf of the emancipation of women. It could not be called pleasant reading, nor is it such as will find acceptation by reasonable minds as a whole. The value of it to the cause which Mrs. Ethelmer has so

THE BOOK WORLD FOR WOMEN AND NURSES—*continued*.

warmly at heart lies in the fact that she has collected the opinions of so many and such weighty minds on the subject. In her desire to see her sex upon a secure platform the authoress ignores the fact that many women hold quite as exalted a position as she would wish for them, and that the individual woman has always had the power to get what she wanted if she set about it the right way, and it is due quite as much to the selfishness of the woman who has all she wants and leaves her less fortunate sisters to fight for themselves, as it is to men, that women as a class are in a position of childhood to-day. Those who are interested in the emancipation question should certainly read Mrs. Ethelmer's book.

### SEPTEMBER REVIEWS.

Miss Emily Crawford contributes to the Contemporary Review a rather stirring view of the style and requirements of "Women Journalists," which has caused considerable amusement among the males of the craft. For women with a taste for "Seeking the bubble reputation. Even in the cannon's mouth," Miss Crawford's highly-coloured pictures will no doubt seem attractive. Quieters souls will perhaps prefer the care of babies, even, if no better may be, of their own.

Westminster Review.

The struggle between the three budgets is well maintained by the "Westminster," the number for September 23rd being specially good. The Great Medicine Man as depicted by Mr. Gould is a masterly piece of work, the face of Dr. Burdon Sanderson being caricatured with force and power. This is a new development of Mr. Gould's powers as an artist, and is full of happy augury for the future. It would be likely to sell largely could the publisher see his way to reproduce the caricature on toned paper. In other respects this issue of the "Westminster" is clever and interesting.

